# Deaf Language Specialists: Delivering Language Therapy in Signed Languages

**DOI:** 10.1093/deafed/enac029

**Published:** 2022-12-12

**Authors:** Joanna Hoskin, Ros Herman, Bencie Woll

**Affiliations:** Department of Language & Communication Division, City, University of London, London, UK; Department of Language & Communication Division, City, University of London, London, UK; Deafness, Cognition and Language Research Centre, University College London, London, UK

## Abstract

Deaf professionals, whom we term Deaf Language Specialists (DLS), are frequently employed to work with children and young people who have difficulties learning sign language, but there are few accounts of this work in the literature. Through questionnaires and focus groups, 23 DLSs described their work in this area. Deductive thematic analysis was used to identify how this compared to the work of professionals (typically Speech and Language Therapists/Pathologists, SLPs) working with hearing children with difficulties learning spoken language. Inductive thematic analysis resulted in the identification of two additional themes: while many practices by DLSs are similar to those of SLPs working with hearing children, a lack of training, information, and resources hampers their work; additionally, the cultural context of language and deafness makes this a complex and demanding area of work. These findings add to the limited literature on providing language interventions in the signed modality with clinical implications for meeting the needs of deaf and hard-of-hearing children who do not achieve expectations of learning a first language in their early years. The use of these initial results in two further study phases to co-deliver interventions and co-produce training for DLSs is briefly described.

In England, around 9% of deaf and hard-of-hearing (DHH) children currently use sign language in some form for interaction with peers and teachers in their educational setting, either on its own or alongside spoken English (Consortium for Research in Deaf Education, 2021 – CRIDE). Of all sign language users, it is estimated that around 6% of DHH children learning British Sign Language (BSL) have specific difficulties with language; this is a similar percentage to that reported for hearing children learning English (Mason et al., 2010). While there has been debate about how to profile children who have unexplained difficulties in their spoken language development (Bishop, 2014), there is agreement that early identification and appropriate intervention is essential (Bercow, 2008). As for other groups with developmental language disorder (formerly known as specific language impairment) (Durkin & Conti-Ramsden, 2010), for DHH children with such problems there is the added impact of language deprivation, resulting from limited language exposure in many families. Language deprivation arises from difficulties establishing communication within families, either because parents use spoken language which their DHH child cannot easily access, or due to a lack of fluent sign language models as a consequence of parents’ limited signing skills leading to long-term effects on children’s mental health and well-being (Gentili & Holwell, 2011).

Speech and Language Therapists/Pathologists (SLPs), alongside colleagues in education and early years provision, provide communication assessment and intervention for children and young people. Training programs for SLPs include assessment and intervention frameworks which guide their work (Bunning, 2004; Roulstone et al., 2012) for use with individuals who have a range of language or communication impairments including: developmental language disorder; language disorder associated with autism or a learning disability; dysfluency/stammer; speech or motor coordination disorders. These frameworks often identify techniques and strategies that are used intuitively by adults when communicating with children. The SLP training enables therapists to have a greater awareness of which skills they are using, why some may be more successful than others, when and how to change strategy, and also to describe this intervention process to others.

The intervention process contains several parts. Bunning (2004) describes the intervention cycle as including techniques such as assessment, diagnosis, goal setting, therapy, and evaluation. She comments that the cycle may not be a linear process, and that aspects of each component may recur at different points in the cycle. She highlights the importance of practitioners from any field sharing a core vocabulary to describe the intervention cycle as this enables the integration of theory and practice, ensuring that practitioners use shared problem solving skills and clinical decision making throughout their interventions.

The format of intervention can vary depending on the setting, goals of intervention and client presentation. Bunning describes five formats: one-to-one, in groups with peers, with an adult other than the therapist, environmental change and advocacy. The selection of a format may be made by the SLP or it may be standardized within the clinical guidelines or established practice for a setting. It may be appropriate to work in one-to-one sessions with a client and provide direct, face-to-face intervention. For other clients, providing intervention with peers in a group may be more suitable. The selection of format may relate to the needs and availability of clients, or setting constraints such as time, staffing levels and the physical environment. Working with another adult to develop communication opportunities and partnerships may also be an effective format for intervention. If the target of intervention is environmental change, sessions with the client may not occur. Instead, the therapist may support others in the client’s environment to make changes that will impact on language and communication. Finally, advocacy-based interventions may be indicated whereby the therapist supports the client to make their own changes in their environment to enhance their effective use of language and communication.

Bunning (2004) further describes intervention techniques that practitioners use to facilitate the therapeutic process between the practitioner and client or other significant stakeholder. For example, engagement techniques are used to support the client or others in engaging with the therapeutic process; modification techniques enable the practitioner to adapt their own use of communication in response to the client’s needs, ensuring the client’s competencies can be identified, and a balanced interaction achieved; feedback techniques are used to enable the client to recognize any behaviors or strategies that promote therapeutic change, and transection techniques facilitate the sharing of information (for example, details of therapeutic input and change) about the client’s language and communication skills, in a timely way with others (the client, families, carers, or other professionals).

These techniques of intervention fit well with a review of practice of professionals working with children with speech, language, and communication needs (Roulstone et al., 2012). Roulstone and colleagues define an intervention broadly as “an action or technique or activity or procedure (or indeed a combination of these) that reflects a shared aim to bring about an improvement or prevent a negative outcome, related to a child’s speech, language and communication skills” (p. 326). They provide two frameworks of intervention useful for comparison to Deaf practitioners’ current practice. The first framework, types of intervention, describes provision of intervention at three different levels relating to a hierarchy of need and provision. At the first level, universal interventions would be available to all children to facilitate language learning and may include access to good language role models and language rich settings. The second level, targeted interventions, are aimed at children who require more support to develop language skills. This support may include small group work with the assistance of trained adults as described by Farmer and Fleur (2006). The third level, specialist interventions, are undertaken for children with the highest level of need for support in learning language. These children often have very specific needs and require use of the language intervention cycle as described above, delivered by a practitioner with additional training in language and intervention.

The second of Roulstone’s frameworks identifies eight categories of interventions used to deliver interventions when working with children or parents or with other practitioners to support them to deliver interventions. These are programmes, intervention activities, principles or approaches, service developed programmes, resources, training, models or theories of intervention and targets of intervention. Depending on the intervention plan, these frameworks can be used with a variety of activities and tasks. These may include everyday personal care tasks such as dressing; daily activities such as games and story books, or practice of specific techniques to enhance a skill, such as breathing and relaxation skills to improve fluency, or motor skill drills to target motor coordination. No such frameworks for therapeutic intervention are available for children who are learning BSL or other sign languages. These frameworks formed the basis for the deductive framework used in the current study (see Table 1).

**Table 1:** Deductive framework based on Bunning (2004) and Roulstone et al. (2012)

Code	Description
Intervention cycle	
Assessment	Tasks and activities identified as being undertaken in order to assess a child’s language
Diagnosis and goal setting	Identification of a language need/deficit or setting a goal/desired outcome linked to a need
Therapy	Providing direct or indirect intervention with the aim of improving the child’s language skills or language use
Evaluation	Measuring, reflecting on and evaluating the success of therapy for a child
Intervention techniques	
Engagement techniques	Techniques used to support the child or others in the therapeutic process
Modification techniques	Techniques used to adapt the language specialist’s own use of communication in response to the child’s, ensuring their competencies can be identified and a balanced interaction achieved (e.g., adapting communication, ascribing meaning, checking interpretation, and understanding)
Facilitation techniques	Techniques used to provide timely support to facilitate language understanding or use (e.g., encouraging contribution, modeling, assisting)
Feedback techniques	Techniques used to promote therapeutic change through feedback (e.g., checking contribution and providing differential, evaluative or summative feedback, acknowledging contributions)
Personal maintenance techniques	Techniques used to recognize and support a child’s needs and behaviors (e.g., emotional, physical, sensory or behavioral acknowledgement or support)
Context maintenance techniques	Techniques used to ensure that the child can engage with the environment and any materials in a positive way (e.g., equipment or setting)
Transection techniques	Techniques used to share information in a timely way with others about the child’s language and communication skills including therapeutic input and change (e.g., gathering information, recording and providing information, advice or instruction, framing, negotiating, explaining or rationalizing)
Intervention format	
1:1	Sessions for therapy including the child and the language specialist only
With peers	Sessions for therapy including the child, one or more peers and the language specialist
With another adult	Sessions for therapy including another adult in order to develop the child or adult’s skills and to develop communication opportunities and partnerships
Environmental change	Supporting others in the environment to make changes
Advocacy	Supporting the child or young person to make their own changes in their environment
Types of intervention	
Universal	Language activities that are available to all children
Targeted	Language activities for children identified as having additional needs (e.g., bilingual, language deprived)
Specialist	Language activities for children with the highest levels of specific language need that involve assessment, diagnosis and delivery of intervention
Categories of intervention	
Programs	A package of activities, arranged in a hierarchical structure, sometimes a published package or reported in a journal
Intervention activities	A discrete activity targeting a specific skill or deficit.
Principles or approaches	Techniques or actions or styles of intervention
Service developed programs	Locally developed, sometimes adapted from published programs, a novel combination of activities, or delivered in a mode particularly suited to local needs.
Resources	Resource names used as shorthand, sometimes referring to an area of language (e.g., narrative) or to an approach (e.g., visual approaches).
Training	Targeting parents or other language specialists to skill them to deliver interventions.
Models or theories of intervention	Theories underpinning interventions.
Targets of intervention	Child’s speech, language and communication underpinning cognitive and processing skills or broader psychosocial aspects of interaction

Researchers have highlighted the importance of DHH children with difficulties learning sign language receiving equitable access to evidence based interventions from trained staff (Herman, Rowley, Mason, & Morgan, 2014; Mann et al., 2014; Quinto-Pozos et al., 2011), comparable to those that underpin spoken language development in hearing children (Law et al., 2010). Although there have been developments in sign language assessments (e.g., Herman et al., 2004, 1999; Mann et al., 2014; Marshall et al., 2014; Woolfe et al., 2010), research on sign language interventions is as yet in its early stages. A few authors have begun to indicate what such interventions may entail, such as interventions focusing on bilingual shared book reading (Andrews et al., 2017; Wolsey et al., 2018) or training children in handshape rhyme awareness (Holcomb & Wolbers, 2020) to enhance language and literacy skills. Holcomb and Wolbers’ (2020) study is of particular interest as it highlights the potential of sign rhyme awareness training in the early identification of language impairment. However, such research is rarely accessed by those working with signing DHH children (Hoskin, 2017).

The assessment of DHH children’s sign language skills and identification of difficulties are complex and best undertaken by language specialists with a range of complementary skills (Marshall & Morgan, 2015). While we now have some tools to assess sign language development, we have more limited information on how to provide intervention once language difficulties are identified and how professionals can work together to share skills. In the UK, staff who deliver interventions to DHH signing children are typically Deaf people with a range of backgrounds working mainly in educational and health care settings alongside professionals such as speech and language therapists, teachers and clinical psychologists. The role of these Deaf practitioners, who we have termed Deaf Language Specialists (DLSs) is particularly important, both linguistically and in terms of identity (Smith & Sutton-Spence, 2005; Sutton-Spence, 2010) for DHH children who use a sign language. Children need to engage in regular interactions with competent adult language users to develop a linguistically rich language. DLSs model Deaf cultural norms and provide models of language and behavior that children can identify with, supporting development of the child’s identity.

However, a challenge facing DLSs in the UK is the limited access to formal professional qualifications in this area. While Deaf people are able to train as teachers, training as SLPs is largely unavailable to them because of the skills required in spoken language and listening on UK SLP courses. The process of designing and delivering language therapy requires high levels of language fluency and specialist knowledge of typical and atypical language development in order to ensure a child’s language skills are assessed and difficulties diagnosed appropriately, and targets are identified for intervention where needed. For DHH children using a sign language, language therapy requires shared practice between different professionals, as SLPs’ skills in language development and disorder need to be complemented by DLSs’ sign language skills.

In England, DLSs work in two main contexts: education and mental health services. The current study focused on the National Deaf Child and Adolescent Mental Health Service (NDCAMHS) which works with children and young people where child, family or school difficulties with language and communication impact on children’s mental health presentation (Wright et al., 2012). Around 25 DLSs currently work within NDCAMHS. Depending on their job role and the aims of intervention, the DLS may meet with a child weekly for assessment, or on other time scales (e.g., daily for some inpatients) to deliver interventions tailored to a child’s care plan.

The scarcity of appropriately qualified Deaf professionals has been highlighted previously by NDCAMHS (Sessa & Sutherland, 2013) and is an area for development. Typically, the role may include “on the job learning” relating to the work they undertake, as available training is generally not accessible to them as DHH sign language users and is not directed at the specific work they do in developing DHH children’s sign language skills. Some DLSs hold qualifications (e.g., in nursing, teaching, clinical psychology) whereas others hold no qualifications, and roles vary from clinical psychologists, family support workers, Deaf consultants, mental health language specialists, and child mental health workers. Where professional qualifications exist, they are recognized by employers and enable clear career progression. Such roles also have defined expectations for ongoing training. For DLSs with skills that do not lead to a nationally recognized professional qualification, career development can be more challenging.

The present study aimed to identify the practices of these Deaf professionals in their work and compare this to SLP practices, to explore the key skills of DLSs, and to identify skills that are needed when delivering language therapy in BSL.

## Research Aims

A three-phase study was designed as a doctoral exploration of this topic. The first phase reported here focused on describing current practice by DLSs and aimed to address the following research questions:

1) How do DLSs currently work with DHH children who have language difficulties?
2) Is the work of DLSs similar to how SLPs work with DHH and hearing children in spoken English?
3) Are there additional aspects of language intervention that are important for DLSs?

A qualitative approach was adopted in order to answer these questions by representing the experiences and actions of people as they encounter, engage, and live through situations (Elliott et al., 1999). Data were collected via questionnaires and focus groups. This first phase was designed to inform subsequent phases of the study and provide useful information for both DLSs and SLPs in their work delivering language therapy in BSL. The second and third phases explored SLTs and DLSs co-delivery of interventions and lead to the co-production of a training package. For information about this later work, the interested reader is directed to Hoskin (2017) and the DOTdeaf project (https://city.ac.uk/dotdeaf).

## Ethics

Approval for the study was obtained from the relevant NHS Research Ethics Committee and local Research and Development Committees for each site. For the questionnaires, a statement was given in the information email informing participants how the information gathered would be used and that completion of the questionnaire implied consent. For the focus groups, consent was gained in writing from each participant for their participation and for video recording of the groups.

## Method

Data collection was of two types: an online questionnaire circulated to DLS networks, followed by three focus groups within NDCAMHS. The use of two methods aimed to broaden the participant group as focus groups could only be held in three geographic locations. The collection of comparable data using both distance and face-to-face data collection tools also allowed comparison of information given anonymously online and in a live group, with colleagues. Deductive and inductive thematic analysis was completed using the framework method (Gale et al., 2013) in order to compare DLSs’ reported practices with those of SLPs and allow further themes to emerge.

### Participants

Thirteen questionnaire participants were recruited via NDCAMHS staff communication channels and special interest groups for teachers and SLPs. Ten focus group participants were recruited via NDCAMHS service communication channels. Information about the groups was circulated and interested language specialists sought their manager’s approval to attend. Participant demographics are shown in Table 2 below.

**Table 2:** Demographics of participants

		Questionnaire	Focus groups
Gender	Male Female	4 9	2 8
Age	26–35 36–45 46–55 56+	1 6 4 2	2 3 4 1
Education and training	School and “on-the-job” Post-school qualifications Graduate or post-graduate	5 3 5	1 4 5
Location	London South East England (excl. London) North East England North West England South West England	8 1 0 2 2	3 2 3 1 1

All 23 participants met the inclusion criterion of working with children who they identified as having language learning difficulties in BSL. Due to the anonymous nature of the questionnaire responses, it is not possible to know if any participants who responded to the questionnaire were also involved in the focus groups. Participants provided information about their own language preferences. For face-to-face interaction almost all participants identified either as bilingual in BSL and spoken English (*n* = 10) or preferring to use BSL only (*n* = 10). One person reported their preferred language was spoken English, while two reported they preferred BSL but used some spoken English. The questionnaire was provided in both BSL and written English formats. All responses returned were in written English, although some questionnaires were completed in written English with the support of BSL/English interpreters. No participants chose to respond in BSL.

Participants were asked to provide details of when and where training about language had been provided. Most language specialists had received some formal training in BSL on courses designed for adult learners of BSL as a second language (http://www.signature.org.uk/british-sign-language), rather than for proficient signers. Although not really appropriate for DHH fluent signers, obtaining these qualifications can have a positive impact on career progression. The largest group (*n* = 11) had a Level 3 qualification. This is equivalent to a modern language exam for high school graduates, or English “A” level. One participant had no formal BSL qualification and five had Level 6 (equivalent to BA level) or BSL tutor qualifications. Information on BSL linguistics is included in the curricula for Level 3 courses and above; at least 16 participants had accessed some training in BSL linguistics. Over half the respondents (*n* = 12) reported that they had no additional training to work with children who had language learning difficulties in BSL. The remaining respondents (*n* = 11) reported attendance at a range of training opportunities, including BSL Production Test training (Herman et al., 2004), National Deaf Children’s Society (NDCS) Family Sign Language training (NDCS, 2022), BSL linguistics courses and in-service training sessions with their employer.

### Questionnaire and focus group procedure

The information email contained the link to the questionnaire where questions were available in written English and BSL video cli ps. Before creating the BSL video clips, the content was discussed with Deaf colleagues in NDCAMHS. A BSL user from this group recorded the agreed information in BSL; this was then translated to English by a qualified BSL/English interpreter and checked by other Deaf colleagues. The final videos were then recorded by an interpreter. Participants had the option to respond in either BSL or English. The questionnaire was available online for three months.

Focus groups were held in BSL and were video recorded for transcription and analysis groups. Because of this, they were kept small and contained three or four participants. Focus groups were led by a BSL user familiar with focus group research.

The questionnaire and focus group questions included four topics: demographics, past training completed by participants, how participants worked with children with language difficulties, and additional ideas or comments on the subject. Questions (Appendix 1) were devised in discussions with two researchers who had worked with Deaf people. They were then trialed with three DLSs who were not study participants. The questions aimed to explore DLSs’ knowledge and understanding of language difficulties and gather examples of their practice as they described it and included case scenarios to facilitate descriptions of practice. DLSs were also asked about their thoughts and processes in order to gather data for subsequent comparison to the work of SLPs (Bunning, 2004; Roulstone et al., 2012). For the focus groups, the questions which explored DLSs’ practice were expanded, illustrated and shown via PowerPoint to aid discussion.

## Data Analysis

All focus group recordings were translated into English by registered English/BSL interpreters and transcribed. Thematic analysis (inductive and deductive) was used to analyze the written English data from the questionnaires and focus groups (Braun & Clarke, 2006; Gale et al., 2013).

The deductive analysis investigated whether themes that have previously been identified as important in the process of delivering language therapy for hearing children in spoken English were also important for DLSs (see Table 1. The framework of codes for the deductive analysis was in five parts: 1) intervention cycle; 2) intervention techniques; 3) intervention format; 4) types of intervention, and 5) categories of intervention. The intervention cycle considered the process of assessment, goal setting, therapy and evaluation. Intervention techniques and format related to how language specialists undertook activities with children and whether this was done individually, in groups or indirectly via other adults. Categories of intervention described the activities that were completed with children. Coding was completed using Daemon Lite software for qualitative data analysis (http://www.provalisresearch.com).

The inductive analysis coded those data which had not been analyzed within the deductive codes and identified themes specific to DLSs working with young people who use BSL. A working analytical framework of codes was developed on the basis of the questionnaire transcripts and then expanded during coding of the focus group data, enabling themes to emerge from the English transcripts.

### Reliability

Guidelines for qualitative research (Yardley, 2000) were applied throughout the study. The first author/lead researcher considered her differing experience as a hearing individual and SLP carefully (“sensitivity to context.”) As part of the process of developing sub-themes, transcripts were coded according to support of the responses for each possible emerging theme. This allowed the data to be traced from initial comments to initial clustering of themes, and to the final structure of themes (Smith et al., 2009). Coding of one focus group and all questionnaire transcripts was conducted by a second SLP. Reliability checks were completed by the lead researcher and another SLP and showed coding agreement of 85% for questionnaire data and 95% for focus group data.

## Results

The deductive analysis results provided information about where DLS practices mapped onto SLP practices and where there appeared to be differences. The inductive analysis results provided examples from the data to support the themes identified.

###  

#### Themes and subthemes identified from deductive analysis

The deductive framework for analysis summarized in Table 1 and is based on the intervention process frameworks (Bunning, 2004; Roulstone et al., 2012) described in the literature review. A discussion of themes identified in focus group and questionnaire responses is given alongside each component of the intervention framework below.

#### Intervention cycle

Assessment, diagnosis and goal setting, therapy and evaluation were all referred to in the questionnaire data and in all focus groups, with assessment discussed most frequently. This is most likely because of research in the UK that has developed BSL assessments, and indeed reference was made to two available standardized BSL assessments. In addition, the use of communication profiles was mentioned. Communication profiles are language and communication screening tools used by professionals such as mental health professionals and DLSs in some UK services. They are a way to assemble information gathered through observations (in home and school settings) and from language and communication sessions with a child in order to profile a child’s communication strengths and development needs. The need for referral for further assessment activities was also raised, e.g.:

- *“We’d ask one of us who has been trained in the BSL productive or receptive test.*”
- *“I’d start by doing a communication profile.”*
- *“They needed a language therapist to do further assessment.”*

#### Intervention techniques

Engagement, modification, transection, and facilitation techniques were mentioned frequently in both questionnaire and focus group data. Personal maintenance techniques were not raised in the questionnaire data but were present in all three focus groups due to the nature of the supportive discussion, e.g.:

- *“Try to match that child’s needs and go at the child’s own pace, not at my pace, so that they are leading me, not that I’m leading them.”*

Feedback techniques came up infrequently in both data sets. Context maintenance techniques were not discussed in questionnaire data and in only two of the focus groups, e.g.:

- *“(I consider) the environment, the room, who’s in the room.*”

#### Intervention format

Reference to working one-to-one with a child was included in questionnaire response data and in all three focus groups., e.g.:

- *“I’m the one who can discuss directly with the child 1:1.”*
- *“I think it’s that you work 1:1.”*

Working with peers and working to achieve environmental change were mentioned in questionnaire responses and in focus groups, e.g.:

- *“Sometimes we’d have two or three in a group. It might be more fun to make sandwiches in a group. They’d help each other and work would be collaborative so we’d compare it so … have they got it right - have they got it wrong - and they’d realize they’d made a mistake on their own.”*
- *“So, we would teach a lot in nursery and they would have nothing when they went home.”*

Working with another adult was not mentioned in the questionnaire data but appeared in all three focus groups, e.g.:

- *“Maybe you could do 2:1 and focus on things in those sessions.”*

Advocacy was not referred to within either dataset.

#### Types of intervention

Three types of intervention: universal, targeted, and specialist, were referred to in the questionnaire data, although specialist interventions were only mentioned by one respondent. Universal and targeted interventions were also discussed by all focus groups, although specialist intervention was not. The challenges of providing incidental language learning were acknowledged, e.g.:

- “*Hearing children have lots of incidental learning as people are playing around them, they’re picking up all the language around them from behind their heads.”*

Differences for children from different language backgrounds were also acknowledged, e.g.:

- “*I’ve noticed that those who are deaf from a deaf family have a very rich level of language. They can sign and that is fine. But from the hearing families there is quite often weakness in different areas of language.”*

For a specialist intervention, one DLS described the process for intervention in more detail:

- *“I would identify a concept or word/sign and ask the child to explain what it means to me. If it’s incorrect, I will explain the meaning clearly or fill in his gaps. I would then expect the child to explain back to me the correct meaning of the concept.”*

#### Categories of intervention

Intervention activities was the most frequently appearing category of intervention in both focus group and questionnaire data. Examples were included of practical activities e.g.:

- *“I’d use pictures of a birthday party for example; it would have a picture of a cake and things that I’d cut from a magazine and there would be one picture that was odd, that wasn’t a birthday party.”*
- *“Something simple without any words, just pictorial, so I can see how their imagination can put a story together and give it back to me.”*

Service-developed programs were not mentioned in either data set but all other categories were: programs, principles or approaches, resources, training, models or theories of intervention, and targets of intervention, e.g.:

- *“Asking them to pretend to be someone else, to see if they are able to do that.”*
- *‘We can say “the child’s nodding and what I do when a child nods: I repeat or I would ask them to repeat back what I was talking about.”*
- *“We can educate staff at the same time; educate staff and the deaf child.”*

To summarize, these results indicate that DLSs consider many of the same approaches as SLPs when working with children with language difficulties. The focus for many DLSs is direct, universal or targeted, one-to-one working with an emphasis on assessment but with little consideration of therapeutic intervention. These findings highlight the potential need for a greater focus on therapeutic intervention after assessment and its evaluation. This is explored further within the discussion section. DLSs also identified the importance of sharing their assessment results with others. The inductive analysis identified some issues that make information sharing a challenging task and these are described below.

### Themes and subthemes emerging from inductive analysis

The inductive analysis resulted in two themes: metalinguistic language and the culture of discussing DHH children’s learning. These themes and sub-themes are described with illustrative quotations below.

#### Metalinguistic language

This theme encapsulates how DLSs use language to discuss language and language difficulties. It highlights some of the challenges inherent in discussing the language difficulties of DHH children and is described in four subthemes: linguistic terminology, English and BSL mixing, foreign language learning, and communication profiles. These subthemes highlight the DLSs’ awareness of the complex nature of their work in contexts where there are differing degrees of professional and family knowledge about sign languages and culture.

Linguistic terms such as “narrative skills”, “timelines”, “handshapes”, “language elements”, turn taking’, and “vocabulary” were used by DLSs. However, while DLSs used these English language linguistic terms in the questionnaire responses, the focus groups provided more insight to DLSs’ understanding of these terms. DLSs used signs for these linguistic terms but there was discussion over which sign linked to which English term. The differences in terminology in BSL used by different DLSs in the focus groups also promoted debate about the meaning of what was being discussed, highlighting the need for more clarification of terminology. One example, timelines, included discussion of narrative sequencing, marking of past tenses, use of space and the development of each of these aspects of sign. Some DLSs were able to explain the terminology they used to their colleagues, whilst others were aware of terms but unable to expand on them.

When the next two sub-themes, English/BSL mixing and foreign language learning, were discussed in the focus groups, DLSs showed an awareness of and some ability to describe the challenges faced by the children they work with due to their multilingual and cultural settings. However, they did not give examples of how to manage these challenges or any specific impacts those difficulties might have on a child’s language or development, beyond it being confusing. The examples below show how complex these issues are to understand and describe.

Examples of English and BSL mixing were given by several DLSs:

- *“If parents are using fluent BSL and the child in school is learning sign supported English and they come home it’s a bit of a mind shift and it can be quite difficult for them to integrate the two.”*
- *“At school he has to speak because he’s in a mainstream school. He goes home and dad’s a fluent BSL signer, and then mom’s talking and signing so he’s exposed to all of them and he’s very confused.”*
- “*We use a visual language in terms of sign language and when children start fingerspelling it is like changing between the two languages.”*

Foreign language learning examples were also in the data set:

- *“I’ve got an example of a child who could be an asylum seeker so they could have some signing ability and I’m sure they would flourish in their home country.”*
- *“Families that have moved from abroad from strong cultural backgrounds and perhaps they (the child) don’t even have any language at all, and then perhaps BSL becomes their (family’s) third language.”*

Finally, DLSs gave examples of using communication profiles as tools to collect and relay information about a child’s language skills and difficulties.

- *“I use the communication profile and look at their conversational skills.”*
- *“We do use communication profiles, and that can be helpful in assessing somebody.”*

The use of communication profiles indicates the beginnings of service-based tools for summarizing and sharing information about a child’s language and communication for DLSs working with children with language difficulties in sign language (Ackroyd et al., 2018).

#### Deaf cultural perspective on DHH children’s language learning

This theme encapsulates two sub-themes: the first, Deaf/proficient sign language models, relates to participants’ understanding of why DHH children may have difficulties with learning language. The second, knowledge, resources or skills relating to working with language difficulties in sign, relates to how language difficulties might be addressed and the barriers DLSs perceive. It should be emphasized that DLSs have to negotiate multilingual and multicultural issues in relation to the development of DHH children, especially as they are often the only adult providing linguistic and social input relating to sign language and Deaf culture. Their responses indicate their sensitivity to and awareness of these issues.

Many children’s language difficulties were ascribed to their lack of access to good language models. In the first sub-theme, DLSs made frequent reference to the lack of Deaf or proficient sign language models:

- “*They don’t get the exposure (to language) from parents…. or school and they’re not getting anything from home.”*
- *“Also, they have less opportunity for that two-way conversation and lots of children are isolated and working with a TA. They haven’t got an opportunity to mix with other deaf peers and so they never learn those narrative skills, they never learn turn taking.”*
- *“I have an example of a child that I visited and they asked, ‘How did you get here?’ and I said ‘Oh, I drove,’ and they replied, “Oh, you’re not allowed. My granddad said deaf people aren’t allowed.” Deaf children can be very concrete and they accept what hearing people have said and that that information is right without any explanation or questioning, so often their view of the world is very limited and they have the view that deaf children aren’t allowed to do things.”*

The benefits of access to good Deaf language models were also mentioned:

- *“And it’s about being creative in that Deaf cultural way.”*
- *“… They (colleagues) ask me to meet that child and I adjust my register to communicate with that child but they can’t communicate with others.”*

Access to adequate language models links to the focus of intervention at a universal or targeted level, that is, intervention for those children with hearing families. Issues related to more specialist interventions were raised in the next sub-theme, where DLSs raised challenges they face in relation to knowledge, resources or skills relating to language difficulties in sign.

- *“It could be that they just don’t understand [BSL], that they can’t access lip reading or they just don’t have a full understanding of English. And again maybe, they could have something like dyslexia or something like that where there are other difficulties in them being able to access the language.”*
- *“Is it affect, linguistic or both? Facial expressions, if they are blank you can see. Quite often children present with very blank facial expressions you don’t …, perhaps they don’t smile. It’s hard to explain really but perhaps they don’t….”*
- *“I don’t use any published resources as they are not appropriate, sufficiently in depth, for the pupils I’m teaching.”*
- *“I can’t think of the word for that but they’re missing some of the features, they’re missing exposure to the full sign language and that’s a problem.”*
- *“I can think of one child who repeated what they were saying, well - copied what I was signing so they weren’t understanding, and they were asking me very inappropriate direct questions like ‘How old are you?’, so they were too direct; they would repeat the same thing again and again; they would go off topic but also insist on not changing the topic and continuing. I don’t know if that was just habit that they would repeat things.”*

A few DLSs commented on how to overcome these challenges:

- *“Get advice from other Deaf Family Support workers, language therapists, BSL tutors - as the more that is shared, the more one learns how to encourage the child to develop their BSL skills.”*

To summarize, in both the questionnaire and focus groups, DLSs reported that they felt able to identify that a child has difficulties with some aspects of language. Their role in managing these difficulties ranged from offering advice to colleagues or families through to planning and delivering individual sessions focused on language. The DLSs report that the language needed to describe children’s difficulties and to explain their interventions was not always available to them. This differs from SLPs who have had training in understanding language development and difficulties, as well as training in explaining their interventions to others. DLSs were also sometimes unsure about how to interpret their observations, where to find appropriate resources to use, how to seek advice, and how to proceed to help the child. For SLPs, clinical supervision is mandatory to maintain registration to practice, and professional development opportunities are widely available. In both the questionnaire and focus group data, DLSs expressed the need for good language models and a Deaf cultural perspective in any support offered to children who have difficulties learning BSL. Some SLPs may be less aware of these needs.

## Discussion

The purpose of this study was to investigate how DLSs currently work with DHH children who have language difficulties and establish whether they work in a similar way to SLPs working with children in spoken English. Additionally, the study aimed to identify any additional aspects of this work that are important for DLSs. The results suggest that there are clear similarities between the work of DLSs and SLPs but that DLSs have limited access to information, training, and resources to support their work. Training about language development in BSL, translanguaging and its impact, and the role of DLSs in this field of work is very limited.

Although researchers have studied the development of BSL (Herman et al., 1999; Marshall et al., 2013; Smith & Sutton-Spence, 2005; Vinson et al., 2008), this information is not always accessible to DLSs nor easily applicable to their practice. More training, tools, and resources are needed to ensure research information can be used with children and their families. Some successful examples of this have happened in the UK such as the I-sign project (Batterbury et al., 2011), which enabled development of the National Deaf Children’s Society’s (NDCS) Family Sign Curriculum (http://www.familysignlanguage.org.uk/mainpage.htm), the Success from the Start materials (NDCS, 2020) and the online assessment portal run by the UCL Deafness Cognition and Language Research Centre (DCAL) (https://dcalportal.org). Aspects of these resources were mentioned by DLSs in this study but more are needed to support the transfer of research into practice.

While there is now a body of research from around the world on assessment in sign languages (Mann & Haug, 2014), researchers in the USA and UK have identified a need for the development of evidence-based interventions to support children with specific language difficulties in sign language (Herman, Rowley, Marshall, et al., 2014; Quinto-Pozos et al., 2011). There is also growing awareness of the need to provide training, supervision, and career development opportunities for Deaf professionals working with children (Gale et al., 2021). A few studies interventions for DHH children are beginning to appear (Andrews et al., 2017; Holcomb & Wolbers, 2020; Mann et al., 2014; Wolsey et al., 2018); however, the DLSs in this study were not aware of any evidence-based practice. By collecting data from these DLSs, practice-based evidence can begin to be established and form the basis of training programs that enable knowledge and good practice to be shared more widely.

### Clinical Implications

The results of the study reported here provide some of the first insights into the work of DLSs. Findings indicate that SLP approaches to intervention can be usefully adapted for sign language, as DLSs currently consider similar processes to SLPs. Training is needed to develop a shared language for DLSs and SLPs to improve co-working and this has until recently not been available to DLSs.

The findings from the present study were confirmed in the second phase of the doctoral project, which observed case studies of SLPs and DLSs co-delivering language therapy and are described in Hoskin (2017). In the third phase, findings were used to develop, deliver, and evaluate a pilot training program aimed at supporting the integration of learning into shared practice, taking account of how Deaf adults prefer to learn. This pilot formed the basis of a successful Erasmus+ funding bid to develop online training across different sign languages, known as the Developing Online Training for Deaf Professionals (DOTDeaf) project (https://city.ac.uk/dotdeaf), culminating in six modules developed in four paired signed and spoken languages: BSL and English, Língua Gestual Portuguesa (LGP) and European Portuguese, Libras and Brazilian Portuguese, and Lengua de Signos Española (LSE) and European Spanish. The modules cover topics highlighted by DLSs in the current and later phases of the study. Further information on module content and the DOTDeaf project is available at https://city.ac.uk/dotdeaf.

The development of a much needed knowledge base and training of DLSs and SLPs for co-working is also supported through an initiative by the National Sensory Impairment Partnership on language planning (Swanwick et al., 2014). This work provides a framework for teachers to develop a language plan in order to support children’s language development for schools. The framework encourages consideration of the child’s language skills and challenges, their communication partners, and all language environments. Although a tool kit in English is already available, BSL resources and training in how to use language plans are yet to be developed.

Numbers of signing DHH children are declining (Consortium for Research in Deaf Education, 2021) and increasingly, research has focused on enabling hearing and spoken language development (Knoors & Marschark, 2012). However, even with improved access to cochlear implantation (National Institute for Health Care and Excellence, 2019), there will always be children who do not receive, or are not suitable for implantation. These children need specifically trained language specialists to help them develop a sign language as a first language during the “critical” or “sensitive” period associated with optimal brain plasticity for first language acquisition (see Bardin, 2012; Hensch & Bilmoria, 2012). There are also other DHH children who, even with technological aids to enable access to sound, do not develop language as expected (Mann et al., 2013). Differential diagnosis of language and learning difficulties is needed for these children. DLSs in the current study highlighted two areas for focus. Firstly, there is a need to consider the language input that children receive to support first language learning in the early years. Secondly, there is a need to deliver language intervention for those with identified additional language learning needs in a sign language. This fits with current research recommendations (Marshall & Morgan, 2015).

Some DLSs reported a mismatch between what was in their job description and what was needed in their work. This has implications for their working practices, training, and supervision, as well as qualifications and remuneration. DLSs’ roles need to be defined for working with children with language difficulties when this is identified as a clinical or educational need. This must then lead to access to training as well as co-working and supervision opportunities with others working with children with language difficulties, whether these are SLPs or specialist teachers.

This paper is the first to report on language therapy in sign language for DHH children and the professionals who deliver it. More evidence from different services and countries is needed. Further research is also needed to develop and evaluate new interventions, and the development and delivery of training and therapeutic resources is a priority. Inequalities in the training available to DLSs must be recognized and addressed. The training developed during later phases of this research (Hoskin, 2017) and in the DOTDeaf project (https://city.ac.uk/dotdeaf) represent a first step towards addressing the urgent need to upskill Deaf professionals working in this area, for the benefit of the children and families with whom they work.

## Appendix

Questionnaire

### Questionnaire in English. Also available online in BSL translation

*Language Therapy in British Sign Language.*

This questionnaire will collect information about how Deaf adults work with children who have language difficulties in BSL. There are four sections. The first asks about you. The second asks about working with children with specific difficulties with BSL. The third section asks you to give any other ideas or information on this topic. The last section asks about your background and training.

*Section 1—Who are you?*

I am collecting information about the people who answer these questions so that I can compare whether the situation is the same across the county. Please tell me about yourself.

a. Male/female
b. Age group 16–25 26–35 36–45 46–55 56+
c. Area in which you work e.g., London, South West England
d. Describe your language preference and use—Only BSL, prefer BSL and use some spoken English, bilingual in spoken English and BSL, prefer spoken English and use some BSL.

*Section 2—What do you do in your work with children?*

I am interested in how people work with children and young people who have difficulties learning BSL. Imagine all the children I will ask you about have people at home and in school who use BSL. Imagine you and your team have identified that a child has difficulties in BSL. The team ask you to work with the child to develop their BSL. Please tell me what you do, what you think is important, what you think about and how you would start work with each child.

What age range of children do you work with?

How do you assess a child’s BSL skills?

Child 1 is 8-years old and has difficulty learning and using new signs. Her sign vocabulary is very small. Please tell me what you would do.

Child 2 is 11-years old. Parents and teachers tell you he does not understand everyday instructions in school or at home. His understanding of BSL is very limited. Please tell me what you would do.

Child 3 is 14-years old. He cannot tell a clear story. When he tells you a story, it is difficult to understand or follow. Please tell me what you would do.

*Section 3—Extra ideas.*

Please tell me about any other strategies and games you use to help children develop BSL.

Tell me about any work you have done to help a child develop their BSL.

*Section 4—your background.*

For the last section, please tell me a bit more about yourself. This will help me know about the background of people working with children who have difficulties in BSL. It will also tell me what training is available.

Do you have educational qualifications? Do you have GCSEs, A levels, degree, other—what are they?

Do you have a formal qualification in BSL? Yes or No.

If yes, what is your qualification in BSL?

Have you done any additional training or been on courses for working with language difficulties in BSL? Yes or No.

If yes, please tell me about these—title, where, when.

Thank you for answering all these questions. If you would like feedback about this project emailed to you, please give an email address.

## References

[ref1] Ackroyd, V., Hayes, R., & Hoskin, J. (2018). The evolution of an innovation; the communication profile - learning and innovation go hand in hand. International Journal on Mental Health and Deafness, 4(1), 37–46. http://www.ijmhd.org/index.php/ijmhd/article/view/48/27 (Accessed 11 August, 2022).

[ref2] Andrews, J. F., Liu, H. T., Liu, C. J., Gentry, M. A., & Smith, Z. (2017). Increasing early reading skills in young signing deaf children using shared book reading: a feasibility study. Early Child Development and Care, 187(3–4), 583–599. 10.1080/03004430.2016.1210135.

[ref3] Bardin, J. (2012). Neurodevelopment: unlocking the brain. Nature Cell Biology, 487(7405), 24–26. 10.1038/487024a. 22763532

[ref4] Batterbury, S., Dickinson, M., Sutherland, H., & West, D. (2011). Signing first : evaluation of I-Sign : the BSL Pilot project. Research report. https://assets.publishing.service.gov.uk/government/uploads/system/uploads/attachment_data/file/181587/DFE-RR137.pdf (Accessed 11 August, 2022).

[ref5] Bercow, J . (2008). The Bercow Report: a review of services for children and young people (0-19) with speech, language and communication needs. Available from: http://dera.ioe.ac.uk/id/eprint/8405.

[ref6] Bishop, D. V. M. (2014). Ten questions about terminology for children with unexplained language problems. International Journal of Language & Communication Disorders / Royal College of Speech & Language Therapists, 49(4), 381–415. 10.1111/1460-6984.12101. PMC4314704 25142090

[ref8] Braun, V., & Clarke, V. (2006). Using thematic analysis in psychology using thematic analysis in psychology. May, 2013, 37–41.

[ref9] Bunning, K. (2004). Speech and language therapy intervention - frameworks and processes. Whurr.

[ref10] Consortium for Research in Deaf Education . (2021). CRIDE 2021 report for England. https://www.batod.org.uk/wp-content/uploads/2022/01/cride-2021-england-report-final.pdf (Accessed 11 August, 2022).

[ref12] Durkin, K., & Conti-Ramsden, G. (2010). Young people with specific language impairment: a review of social and emotional functioning in adolescence. Child Language Teaching and Therapy, 26(2), 105–121. 10.1177/0265659010368750

[ref13] Elliott, R., Fischer, C. T., & Rennie, D. L. (1999). Evolving guidelines for publication of qualitative research studies in psychology and related fields. The British Journal of Clinical Psychology / the British Psychological Society, 38(Pt 3), 215–229. 10.1348/014466599162782. 10532145

[ref14a] Farmer, M., & Fleur, G . (2006). The Talking Table. In Clegg, J., & Ginsborg, J. (Eds.), Language and Social Disadvantage - Theory into practice. United Kingdom: John Wiley & Sons Ltd. pp. 165–176.

[ref14] Gale, E., Berke, M., Benedict, B., Olson, S., Putz, K., & Yoshinaga-Itano, C. (2021). Deaf adults in early intervention programs. Deafness and Education International, 23(1), 3–24. 10.1080/14643154.2019.1664795.

[ref15] Gale, N. K., Heath, G., Cameron, E., Rashid, S., & Redwood, S. (2013). Using the framework method for the analysis of qualitative data in multi-disciplinary health research. BMC Medical Research Methodology, 13(1), 117. 10.1186/1471-2288-13-117. 24047204 PMC3848812

[ref16] Gentili, N., & Holwell, a. (2011). Mental health in children with severe hearing impairment. Advances in Psychiatric Treatment, 17(1), 54–62. 10.1192/apt.bp.109.006718.

[ref17a] Hensch, T. K., Bilimoria, & P. M. (2012). Re-opening Windows: Manipulating Critical Periods for Brain Development. Cerebrum. 11. Epub. PMID: 23447797; PMCID: PMC3574806. PMC3574806 23447797

[ref17] Herman, R. C., Grove, N., Holmes, S., Morgan, G., Sutherland, H., & Woll, B. (2004). Assessing British Sign Language Development: Production Test (Narrative Skills). City University.

[ref18] Herman, R. C., Holmes, S., & Woll, B. (1999). Assessing BSL Development: Receptive Skills Test. England: Forest Books.

[ref19] Herman, Rosalind, Rowley, K., Marshall, C., Mason, K., Atkinson, J. R., Woll, B., & Morgan, G. (2014). Profiling SLI in deaf children who are sign language users. In David Quinto-Pozos (Ed.), Multilingual Aspects of Signed Language Communication and Disorder (pp. 45–69). Multilingual Matters. 10.21832/9781783091317-005.

[ref20] Herman, R., Rowley, K., Mason, K., & Morgan, G. (2014). Deficits in narrative abilities in child British Sign Language users with specific language impairment. International Journal of Language & Communication Disorders, 49(3), 343–353. 10.1111/1460-6984.12078. 24617640

[ref21] Holcomb, L., & Wolbers, K. (2020). Effects of ASL rhyme and rhythm on deaf children’s engagement behavior and accuracy in recitation: evidence from a single case design. Children, 7(256), 1–31. 10.3390/children7120256. PMC7761000 33255943

[ref22] Hoskin, J. H. (2017). Language Therapy in British Sign Language: a study exploring the use of therapeutic strategies and resources by deaf adults working with young people who have language learning difficulties in British Sign Language (BSL). Unpublished Doctoral dissertation. UCL (University College London).

[ref23] Knoors, H., & Marschark, M. (2012). Language planning for the 21st century: revisiting bilingual language policy for deaf children. Journal of Deaf Studies and Deaf Education, 17(3), 291–305. 10.1093/deafed/ens018. 22577073

[ref24] Law, J., Lee, W., Roulstone, S., Wren, Y., Zeng, B., & Lindsay, G. (2010). “What Works”: interventions for children and young people with speech, language and communication needs: Technical Annex. London: Department for Education Research Report.

[ref25] Mann, W., & Haug, T. (2014). Mapping out guidelines for the development and use of sign language assessments: some critical issues, comments and suggestions. In David Quinto-Pozos (Ed.), Multilingual Aspects of Signed Language Communication and Disorder (pp. 123–139). Multilingual Matters. 10.21832/9781783091317-008.

[ref26] Mann, W., Peña, E. D., & Morgan, G. (2014). Exploring the use of dynamic language assessment with deaf children, who use American Sign Language: Two case studies. Journal of Communication Disorders, 52, 16–30. 10.1016/j.jcomdis.2014.05.002. 24908340

[ref27] Mann, W., Roy, P., & Marshall, C. (2013). A look at the other 90 per cent: investigating british sign language vocabulary knowledge in deaf children from different language learning backgrounds. Deafness & Education International, 15(2), 91–116. 10.1179/1557069X12Y.0000000017.

[ref28] Marshall, C., Mason, K., Rowley, K., Herman, R., Atkinson, J., Woll, B., & Morgan, G. (2014). Sentence repetition in deaf children with specific language impairment in british sign language. Language Learning and Development, 1–15.

[ref29] Marshall, C., & Morgan, G. (2015). Investigating sign language development, delay, and disorder in deaf children. In M. Marschark & P. E. Spencer (Eds.), The Oxford Handbook of Deaf Studies in Language (pp. 1–27). Oxford University Press.

[ref30] Marshall, C. R., Rowley, K., Mason, K., Herman, R., & Morgan, G. (2013). Lexical organization in deaf children who use British Sign Language: evidence from a semantic fluency task. Journal of Child Language, 40(1), 193–220. 10.1017/S0305000912000116. 22717181

[ref31] Mason, K., Rowley, K., Marshall, C. R., Atkinson, J. R., Herman, R., Woll, B., & Morgan, G. (2010). Identifying specific language impairment in deaf children acquiring British Sign Language: implications for theory and practice. British Journal of Developmental Psychology, 28(1), 33–49. 10.1348/026151009X484190. 20306624

[ref10a] National Deaf Children’s Society . (2020). Success from the start: A developmental resource for families of deaf children 0-3. https://www.ndcs.org.uk/documents-and-resources/success-from-the-start-a-developmental-resource-for-families-of-deaf-children-aged-0-to-3/ (Accessed 11 August, 2022).

[ref35a] National Deaf Children’s Society . (2022). Family Sign Language. https://www.ndcs.org.uk/information-and-support/language-andcommunication/sign-language/family-sign-language/ (Accessed 11 August, 2022).

[ref34a] National Institute for Health Care and Excellence . (2019). Cochlear implants for children and adults with severe to profound deafness. https://www.nice.org.uk/guidance/ta566 (Accessed 11 August, 2022). 40902019

[ref35] Quinto-Pozos, D., Forber-Pratt, A., & Singleton, J. (2011). Do developmental communication disorders exist in the signed modality? Perspectives from professionals. Language, Speech, and Hearing Services in Schools, 42, 423–443. 10.1044/0161-1461(2011/10-0071). 21844402

[ref36] Roulstone, S., Wren, Y., Bakopoulou, I., & Lindsay, G. (2012). Interventions for children with speech, language and communication needs: an exploration of current practice. Child Language Teaching and Therapy, 28(3), 325–341. 10.1177/0265659012456385.

[ref37] Sessa, B., & Sutherland, H. (2013). Addressing mental health needs of deaf children and their families: the National Deaf Child and Adolescent Mental Health Service. The Psychiatrist, 37(5), 175–178. 10.1192/pb.bp.112.038604.

[ref38] Smith, S., & Sutton-Spence, R. (2005). Adult–child interaction in a BSL nursery — getting their attention! Sign Language & Linguistics, 8(1), 131–152. 10.1075/sll.8.1.07smi.

[ref39] Sutton-Spence, R. (2010). The role of sign language narratives in developing identity for deaf children. Journal of Folklore Research, 47(3), 265–305. 10.2979/jfolkrese.2010.47.3.265.

[ref40] Swanwick, R., Simpson, K., & Salter, J. (2014). Language Planning in Deaf Education Guidance for Practitioners by Practitioners. The National Sensory Impairment Partnership.

[ref41] Vinson, D. P., Cormier, K., Denmark, T., Schembri, A., & Vigliocco, G. (2008). The British Sign Language (BSL) norms for age of acquisition, familiarity, and iconicity. Behavior Research Methods, 40(4), 1079–1087. 10.3758/BRM.40.4.1079. 19001399

[ref43] Wolsey, J. L. A., Clark, M. D., & Andrews, J. F. (2018). ASL and English bilingual shared book reading: an exploratory intervention for signing deaf children. Bilingual Research Journal, 41(3), 221–237. 10.1080/15235882.2018.1481893.

[ref44] Woolfe, T., Herman, R., Roy, P., & Woll, B. (2010). Early vocabulary development in deaf native signers: a British Sign Language adaptation of the communicative development inventories. Journal of Child Psychology and Psychiatry, and Allied Disciplines, 51(3), 322–331. 10.1111/j.1469-7610.2009.02151.x. 19843318

[ref45] Wright, B., Walker, R., Holwell, A., Gentili, N., Barker, M., Rhys-Jones, S., … Moore, K. (2012). A new dedicated mental health service for deaf children and adolescents. [References]. *Advances in Mental*. Health, 11(1), 95–105. 10.5172/jamh.2012.11.1.95.

[ref46] Yardley, L. (2000). Dilemmas in qualitative health research. Psychology & Health, 15(2), 215–228. 10.1080/08870440008400302.

